# Randomized Trial to Improve Primary Care Patient Management and Patient Outcomes Using a Drug–Drug Interaction Test: Confirmation of the DECART Simulated Patient Clinical Utility Trial Results

**DOI:** 10.3390/diagnostics11071266

**Published:** 2021-07-15

**Authors:** John Peabody, Joshua Schrecker, Rebecca Heltsley, David Paculdo, Enrico de Belen, Diana Tamondong-Lachica, M. Czarina Acelajado, Othman Ouenes, Trina Kennedy, Elaine Jeter

**Affiliations:** 1 College of Medicine, University of California, San Francisco, CA 94143, USA; 2 Fielding School of Public Health, University of California, Los Angeles, CA 90095, USA; 3 QURE Healthcare, San Francisco, CA 94133, USA; dpaculdo@qurehealthcare.com (D.P.); edebelen@qurehealthcare.com (E.d.B.); dlachica@qurehealthcare.com (D.T.-L.); czarina@qurehealthcare.com (M.C.A.); oouenes@qurehealthcare.com (O.O.); 4 Aegis Sciences Corporation, Nashville, TN 37228, USA; joshua.schrecker@aegislabs.com (J.S.); rebecca.heltsley@aegislabs.com (R.H.); elaine.jeter@aegislabs.com (E.J.); 5 TJK Consulting, Sandpoint, ID 83864, USA; trinajkennedy@gmail.com

**Keywords:** primary care, drug–drug interactions, clinical utility, internal medicine, family medicine

## Abstract

Drug–drug interactions (DDIs) are a serious problem in the healthcare system, leading to excess healthcare utilization and costs. We conducted a second prospective randomized, controlled trial to further establish the real-world clinical utility of a novel assay that objectively identifies potentially serious DDIs in real-world patients. Re-recruiting primary care physicians (PCPs) from our first randomized, controlled, simulated-patients study on DDIs, we experimentally introduced a definitive, urine-based mass spectrometry test intervention that the physicians could use when caring for their eligible patients. Patients were eligible if taking four or more prescription medications or suspected of taking other non-prescribed substances with potential medication interactions. The primary outcome was whether DDI testing changed clinical care. We explored a secondary outcome to see if the change in practice improved symptoms in patients with potential DDIs. A total of 169 control and 162 intervention patients were enrolled in the study, and their medical records were abstracted. In real-world patients, intervention physicians identified and/or treated a DDI at 3.0x the rate in their patient population compared to controls (21.6% vs. 7.1%, *p* < 0.001). Intervention physicians were more likely to discontinue or adjust the interacting agent compared to controls (62.9% vs. 8.3%, *p* = 0.001), and patient-reported symptoms also significantly declined (29.6% vs. 20.1%, *p* = 0.045). These results were nearly identical to concurrent measurements that used simulated patients, wherein intervention was more likely to both make a DDI diagnosis (56.3% vs. 21.6%, *p* < 0.001) and stop the interacting medications (58.3% versus 26.6%, *p* < 0.001). Bringing a new diagnostic test to market, particularly for an under-recognized clinical problem, requires robust data on both clinical validity and clinical utility. The results of this follow-up study showed that the use of DDI testing in real-world patients significantly improved (1) primary care patient management of drug interactions and (2) patient outcomes.

## 1. Introduction

Drug–drug interactions (DDIs) are underdiagnosed, accounting for more than 30% of all adverse drug events (ADEs) [1,2,3], which cost the collective healthcare system an estimated USD 30.1 billion [4,5,6,7]. The number of patients taking five or more prescription drugs has more than doubled between 1988 and 2018 [8], while past research confirms that the risk of an ADE or DDI increases with polypharmacy [9], highlighting the urgent need for a DDI test with demonstrable clinical utility.

Aegis Sciences Corporation has analytically and clinically validated a urine- or oral-fluid-based DDI test. The DDI test (InterACT Rx^TM^) utilizes a sensitive and specific liquid chromatography–mass spectrometry-based method to detect 150 unique interacting substances. Detected substances are then referenced to a comprehensive DDI information database that reports interactions between the ingested substances, which the ordering physician can use to adjust treatment. The urine-based test detects prescription and non-prescription substances that are commonly prescribed or ingested by patients with common comorbid medical conditions and are capable of impacting prescription medication pharmacokinetics (e.g., drug absorption and/or metabolism) or pharmacodynamics. In addition to providing objective information regarding ingested substances, the test results also describe the interaction severities (e.g., moderate, severe, contraindicated) and provide clinical descriptions of the interactions.

In May 2018, among a nationwide sample of primary care physicians (PCPs), we performed a simulated-patient study to demonstrate the clinical validity of DDI testing. Using validated Clinical Performance and Value (CPV^®^) simulated-patient vignettes [10,11], we conducted a randomized, controlled, cross-sectional study, known as the first DDI Effectiveness and Clinical Awareness Randomized Controlled Trial (DECART1) [12,13]. DECART1 showed that DDI-related preventive care practices were worrisome and wanting [12]. Despite > 99% of the national representative sample of PCPs reporting routinely performing medication reconciliations, serious and identifiable DDIs were detected in only 15% of symptomatic CPV simulated patients [12]. When DDI testing was experimentally introduced to intervention PCPs in the sample frame, the results were dramatic: the DDI diagnosis rate among simulated patients increased from 16.7% to 56.7% (3.4×), stopping the interacting drug increased from 21.3% to 60.9% (2.9×), and counseling of patients with DDIs increased from 6.1% to 21.4% (3.5×) [13].

Herein, we report on the follow-up DECART (DECART2) to confirm that this dramatic effect is translated into PCPs’ practice and their patients. We wanted to determine whether the test (a) increased identification of DDIs by PCPs in their real-world patients, (b) led to a change in their patients’ medications or, as a secondary outcome, (c) improved their patients’ symptom resolution, compared to patients of control PCPs who were not given the DDI test.

## 2. Materials and Methods

Between November 2019 and November 2020, we collected DECART2 study data. The goal of the study was to provide additional evidence of the clinical utility of DDI testing. We first re-recruited control and intervention PCPs from DECART1, and used CPVs to re-measure the clinical practice change associated with the introduction of DDI testing. We then introduced an objective DDI test into the real-world setting among the subset of DECART1 intervention PCPs who agreed to participate. We then secured data on practice patterns from the medical records of both intervention and control DECART2 groups. The outcome measures were the change in DDI diagnostic accuracy and changes made in practice by the intervention group compared to the controls.

### 2.1. Study Design and Data Sources

DECART2 is a prospective, randomized, controlled trial that collected data over 13 months. DECART2, like DECART1, experimentally introduced DDI testing to roughly half of the study’s participants. In DECART2 we leveraged the original randomized sample frame created in DECART1 to re-enroll the PCPs into the new study. The intervention group was then given the DDI test, and we asked them to use the test in their clinical practice on their eligible patients. The control group used the same patient enrollment criteria, but was not given the test.

### 2.2. Ethics

This study was conducted in accordance with ethical standards and approved on 24 September 2019 by the Advarra Institutional Review Board (IRB) (Columbia, MD, USA). The trial is also listed on clinicaltrials.gov (accessed on 2 March 2021) (NCT03581994). Informed consent was obtained from all participants.

### 2.3. Physician Selection

All 313 physicians who participated in the initial DECART1 trial were invited to participate in DECART2. The eligibility criteria for PCPs in DECART1—and thus, the eligibility criteria for DECART2—were (1) board-certified in either internal medicine or family medicine, (2) in a non-academic setting, (3) between 2 and 30 years of post-residency practice, and (4) an active panel of over 500 patients with an adult patient load of more than 50%. 

In total, 88 physicians (47 intervention and 41 control) agreed to participate in DECART2. Of the 88, 46 dropped out due to time constraints or COVID-19-pandemic-associated changes to their practice. The remaining 42 (21 in each study arm) submitted complete records for their eligible patients. The 46 dropouts were compared to the 42 participants who enrolled patients into the study. We found no significant differences in gender, age, or practice characteristics between the two groups (*p* > 0.05 for all).

### 2.4. Patient Selection

All participating physicians were asked to recruit 6–10 new or returning patients who met pre-specified inclusion criteria. Eligible patients had to fulfill the same criteria as the CPV patient simulations—namely, (1) receiving pharmacological treatment for a behavioral health condition (e.g., depression, anxiety, schizophrenia, etc.) or chronic pain; (2) taking 4 or more drugs; (3) having new and/or undiagnosed clinical symptoms; (4) being at least 21 years old; and (5) their physician being concerned about non-compliance with their medications or the use of non-prescribed substances—such as opioids, alcohol, or OTC supplements. The DDI test was ordered by the PCP after performing initial history and physical examination, as part of other diagnostic workups that the patient might need during their visit.

Participating PCPs enrolled and delivered charts on 174 intervention and 169 control patients. Twelve intervention charts were excluded due to absent follow-up records. In total, 162 intervention and 169 control patients had the medical records of their initial visit—and all subsequent visits for up to 3 months—abstracted and reviewed.

In compliance with the IRB approval for the study, any patients meeting these criteria and selected by their intervention physicians first consented to submit a urine sample for DDI testing. Urine samples for these patients were collected by the intervention physicians, who submitted the samples directly to the laboratory for analysis. Test results were returned within 3–4 days.

### 2.5. Data

The first dataset was a set of CPV vignettes—simulated patients aged 30–75, who presented to outpatient PCPs with clinical conditions putting them at risk for a moderate (defined as an interaction which results in exacerbation of the patient’s condition) or severe (defined as either a contraindicated or major interaction with a potentially life-threatening or other major adverse reaction) DDI. The CPV simulated cases are validated measures of actual provider practice, controlling for case-mix variation, and widely used to determine clinical practice changes (clinical utility) [10]. These were originally used in DECART1 [12,13], and these same vignettes were used in the present DECART2 study to re-measure physician practice patterns. The nine CPV patient simulations in both DECART studies were possible use cases representing a variety of drug interaction presentations identified in the history (symptoms), signs, and/or drug profiles (including prescribed medications, over-the-counter medications, and foods). In DECART2, PCPs took care of three CPVs that they had not taken care of before in DECART1 [12]. All participating DECART2 providers retook the simulations in order to ensure (1) that there were no intervening secular changes in practice that would have obviated the need for the test, and (2) that the DDI test still had clinical utility within the intervention group, thus reconfirming the DECART1 results. Clinical care for all cases was tracked over five separate domains: (1) medical history, (2) physical examination, (3) clinical testing, (4) diagnosis, and (5) pharmacotherapy changes and follow-up recommendations. Control physicians were unable to order a DDI test; the intervention group was provided DDI test results for each case. Participant responses were scored against explicit evidence-based criteria by two expert, blinded physician reviewers, and a third expert physician who served as an adjudicator in case of disagreement. Results are presented as a percentage score between 0% and 100% of correct care items identified.

The second data source was abstracted, anonymized patient charts of all eligible patients who consented to participate in the study and whose records were available for abstraction. Both groups entered their patient data into a de-identified abstraction form. All chart entries were from the time of enrollment, which was concurrent with the DDI testing of urine samples in the intervention group, until three months after that first visit. The follow-up period allowed for sufficient time to pass for the DDI test results to be sent back to the intervention physicians, and for clinical action or interventions to be undertaken. We measured the differences in downstream care between the control and intervention groups.

The de-identified data was sent to a third-party abstraction team that scored the charts against explicit criteria, creating a dataset for analysis. In compliance with section 164.514 of the National Institutes of Health HIPAA Privacy Rule, for review and analysis, the data were only linked to physicians via a unique identification number.

### 2.6. Intervention

During DECART1, physicians were randomized into either the control group, which offered standard care, or the intervention group, which offered standard care plus DDI test educational material and results in the second round of data collection. In DECART2, the actual DDI test, as currently offered in the market, was introduced for intervention group patients. The full list of substances, tested by definitive mass spectrometry, is given in Supplementary Table S1. All intervention arm participants also received refresher educational materials describing the DDI test, consisting of (1) a short information webinar, (2) one case study, (3) a sample test report, (4) an overview brochure on the DDI test, and (5) a background document on DDIs. Intervention arm physicians were sent the DDI test results from submitted urine samples, at no cost to the physician or the patient.

### 2.7. Analysis

The primary outcome was whether the introduction of DDI testing improved clinical practice through appropriate changes in medical management. We measured practice changes in two ways: using simulated patients through the CPVs, and in the real world using abstracted medical records. We compared the intervention patients who received any DDI-related diagnosis or treatment against control patients. For intervention, a DDI was present if a moderate or severe interaction was reported in DDI test results. For control, a DDI was counted if noted in the physicians’ charts. Specifically, we examined differences in DDI diagnosis, defined as either making the diagnosis or treating for a DDI by stopping the interacting drug or counseling on DDIs. Secondarily, we wanted to see whether there was a significant difference in symptom resolution caused by the DDI, and whether there was a difference in hospitalizations or emergency department (ED) visits. We also compared the DDI-induced practice change using the CPVs to the changes found in the medical records. We used the chi-squared test for single binary independent variables, and logistic regression for multivariate modeling for categorical dependent outcomes. Student’s *t*-test was performed for analyses involving continuous outcomes. All analyses were conducted in Stata 14.2 (StataCorp, LLC, College Station, TX, USA).

## 3. Results

### 3.1. Physician Characteristics

Comparing the physician characteristics, we found no significant differences between the two groups in terms of age, gender makeup, years of experience, or practice location or type (*p* > 0.05 for all, Table 1).

### 3.2. CPV Vignette Scores

Physicians in the intervention group were more than twice as likely to make a DDI diagnosis compared to the control group (56.3% vs. 21.6%, *p* < 0.001), as well as to stop the interacting medications (58.3% vs. 26.6%, *p* < 0.001).

In addition to being 2.6 times more likely to make the DDI diagnosis, intervention physicians were significantly more likely to note the specific DDI in the CPVs (16.4% vs. 3.6%, *p* < 0.001), and to advise their patients about potentially severe DDIs (29.2% of cases vs. 8.3%, *p* = 0.001).

### 3.3. Patient Characteristics

There were no statistically significant differences between the patients in the two groups by gender (50.6% male for intervention vs. 52.7% for control, *p* = 0.710) or average age (59.1 for intervention vs. 58.5 for control, *p* = 0.590) (Table 2). There was also no difference in suspected compliance to their prescription medications (76.0% for intervention vs. 72.2% for control, *p* = 0.699), use of excessive alcohol or non-prescribed controlled medications, including opioids (47.5% for intervention vs. 50.3% for control, *p* = 0.615), or use of other interacting substances—such as supplements, foods, and over-the-counter medications—identified as possible risk factors for DDIs (54.9% for intervention vs. 52.1% for control, *p* = 0.601).

### 3.4. Patient Results

Real-world patients who consented to participate had the same selection criteria as the simulated CPV patients, as noted in the Methods section above.

Physician management of drug interactions was examined in order to determine which factors were significant in detecting or treating DDIs. In the CPVs, only access to the objective DDI test was related to changes in practice, defined as diagnosing or treating DDIs more often (O.R. 5.8; 95% C.I. 2.6–12.8). The same multivariate regression model was used to evaluate the management of the consenting patients, and here we also found that only the use of the DDI test improved DDI detection or treatment (O.R. 4.1; 95% C.I. 2.0–8.5).

Overall, 21.6% (35/162) of intervention patients and 7.1% (12/169) of control patients were diagnosed/treated by their doctors for moderate and severe DDIs—a threefold difference between the two groups (Table 3). Interestingly, of the 35 intervention and 12 control patients who had a DDI recognized, in both groups only around half (16 of the 35 intervention patients and 6 of the 12 of the control patients) had a DDI diagnosis explicitly in their charts. In the rest of the recognized cases, although the DDI was not recorded in the chart, it was treated.

Physician treatment for DDIs was also different between the two groups; intervention physicians discontinued/adjusted the interacting agent in 63% of the 35 identified intervention patients, versus just 8% in the 12 identified control patients (*p* = 0.001). For cases not related to prescribed medications—such as when alcohol or an over-the-counter substance was the interacting agent/suspected interacting agent—there was no difference between counseling by the intervention physicians compared to controls (49% vs. 58%, *p* = 0.559).

### 3.5. Analysis of DDI Test Results

When the DDI test results were evaluated, 127 of the 162 eligible patients (78.4%) tested positive for a DDI, with an average of 2.3 + 1.2 interactions per positive test result. Of the 127 patients with any DDIs identified, 38 test results reported a severe DDI (29.9%), and among these 38 patients there was an average of 1.1 + 1.0 DDIs per test-positive patient. 

Not all severe DDIs detected by the urine test were acted on by the intervention physicians. Of the 38 patients in the intervention group with severe DDIs found by urine testing and recorded on their charts, 31 were DDI diagnosed/treated according to their charts, but 7 were not. By comparison, in the control group only 10 severe DDIs were noted in the chart and treated. 

The vast majority of severe DDIs detected in this study involved one or more of the following four classes of substances: opioids (44%), benzodiazepines (27%), alcohol (23%), and behavioral medications (antipsychotics 13%, antidepressants 10%, and anxiolytics 5%). A benzodiazepine–opioid interaction was present in 12.8% of all severe DDI samples, followed closely by behavioral medicine interactions in 11.5%, alcohol–opioid interactions in 11.5%, and alcohol–benzodiazepine interactions in 10.3%. The opioid–benzodiazepine–alcohol trio of interactions, for example, is considered severe because their combined use enhances the effects of the individual drugs, leading to side effects such as excessive sedation, respiratory depression, and death. Behavioral medication interactions (such as antidepressants and antipsychotics) could lead to QT prolongation and tachycardia, provoking possible loss of consciousness or seizures. Other less frequent but important DDIs in this population lowered the effectiveness of medical therapy—such as eating grapefruit (3.8%), which can block CYP3A4 and increase the levels of circulating statins and other prescribed medications.

### 3.6. Resolution of Symptoms and Hospitalizations/ED Visits

We wanted, as a secondary outcome, to see whether there was any improvement in the symptoms patients were reporting when the DDI test was used. Compared to patients in the control group, physician-recorded symptoms improved more for the intervention group than for the control group, with 48 of 162 intervention patients (29.6%) improving versus 34 of 169 control patients (20.1%) (*p* = 0.045). This improvement likely underestimated the benefits of DDI detection, since symptom reduction is only one manifestation of a DDI. For example, some DDIs are biochemical and, while important, do not produce symptoms; other DDIs may not be reported by patients, or symptoms may be attributed to the underlying condition and not to the DDI. 

Analyzing the subset of 48 intervention and 34 control patients with symptom resolution, 18 of 48 intervention patients (37.5%) who saw symptom resolution were also diagnosed/treated for a DDI, compared to only 4 of 34 control patients (12.1%) (*p* = 0.012).

We further examined whether DDI detection and treatment led to fewer hospitalizations or reduced ED utilization, and while trending towards less utilization for the intervention group, this did not reach significance (12 of 162 (7.4%) in intervention versus 19 of 169 (11.2%) in control; *p* = 0.231).

### 3.7. Comparison of Real-World and CPV Results

We found a high degree of agreement between the DECART2 CPV and real-world practice results. In the simulated CPV study, intervention PCPs—when equipped with the DDI test results—made a DDI diagnosis 2.6 times more frequently, and were 2.2 times more likely to stop an interacting substance than control PCPs (*p* < 0.001 for both, Table 1). In the real-world DECART2 study, intervention physicians were 3.0 times more likely to identify a DDI compared to controls (*p* < 0.001), and 7.6 times more likely to stop the interacting substance (*p* = 0.001) (Table 3).

## 4. Discussion

DDIs are harmful to patients, and a preventable driver of healthcare costs. The major DECART2 findings are that physicians who used the DDI test in their practice were three times (21.6% vs. 7.1%) more likely to identify the DDI and, once it was identified, were seven times (62.9% vs. 8.3%) more likely to change patients’ pharmacotherapy regimen. Intervention patients’ symptoms were also three times more likely to resolve when a DDI diagnosis or treatment was started (37.5% vs. 12.1%). These results were nearly identical to the published findings from DECART1, conducted 18 months earlier, wherein the intervention group made the DDI diagnosis 56.7% of the time and stopped the interacting medication 60.9% of the time.

A major finding, not fully appreciated in the original DECART1, is the magnitude of DDIs in patients taking four or more medications, and the inadequacy of mitigating the risks of a DDI if the diagnosis is missed. In DECART2, DDIs were detected 78.4% of the time among this population of patients, and when found, the DDI test results showed on average 2.3 DDIs per patient, 1.1 of which were severe. 

When DDIs were diagnosed and treated, physicians recorded that their patients got better. The noted patients’ benefits, which were only determined if they were noted in the patient’s chart, likely overlooked benefits attributed to other causes, such as symptomless biochemical changes, clinical improvement from other causes, and under-recording. Notwithstanding, DDI-related symptoms resolved 29.6% of the time in the intervention group compared to only 20.1% in the control group (*p* = 0.045), and although DDI detection and treatment trended towards fewer hospitalizations and reduced ED utilization, this result was likely underpowered, and did not reach statistical significance (7.4% vs. 11.2%; *p* = 0.231).

Gathering sufficient data to prove clinical utility has proved to be costly and slow for makers of novel diagnostic tests [14]. Payers and regulators, who ultimately decide on coverage and reimbursement, likewise want new diagnostic and therapeutic products to be introduced [15] but need high quality data showing that the test changes clinical practice and improves patient health without introducing more costs [16]. To overcome these temporal and financial barriers, a newer, validated approach to large multicenter randomized controlled trials is increasingly being used to secure coverage and reimbursement [17]. The newer approach uses validated patient simulations in randomized clinical trials, which are lower cost and less time-intensive than large, multicenter, patient-based trials. CPVs, which are extensively validated simulated patients, [10,11,17,18] have important advantages over real-world patients: (1) they specify the use case and eliminate patient heterogeneity; (2) they focus on whether the test changes physician behavior; and (3) they can generate high-quality data in short periods of time. Despite CPVs’ ability to track actual clinical practice in multiple settings among a legion of clinical conditions, for some, the question remains as to how well these changes in CPVs translate into specific changes in clinical practice and better outcomes. 

In this study, we had another chance to rigorously test whether the clinical utility—first established using simulated patients—translated into real-world clinical practice change and better patient outcomes for an important new diagnostic test. In the simulation-only DECART1, the evidence was overwhelming: PCPs caring for simulated patients improved their diagnostic and therapeutic performance dramatically when introduced to a DDI test. PCPs identified potentially severe DDIs in only 15% of patients [12] when DDI testing was not utilized. When given access to the DDI test results, intervention PCPs were able to identify DDIs three and a half times more often, and stopped the interacting drug at three times the rate of their care at baseline. For many, this would be adequate, high-level evidence of clinical utility. In the present study, we confirmed these findings in the simulated patient study.

There have been other examples of real-world patient studies confirming simulated CPV studies [17]. Therein, we examined the clinical utility of a blood-based protein assay to quantify the potential for colorectal cancer in patients with elevated risk, and ascertained whether physicians with this information were more likely to screen their patients for cancer. In the simulated CPV study, physicians were 1.3 times more likely to order a diagnostic colonoscopy for their higher-risk patients (*p* < 0.001), which was consistent with the subsequent real-world patient study, where these same physicians were 4.6 times more likely to order a diagnostic colonoscopy (*p* = 0.027). We note that not only are the simulated results consistent with the real-world results, but the simulated study conservatively underestimates the impact on practice, as it did in the DECART2 study.

The introduction of a DDI test, as shown now in two prospective studies, addresses an enormous and underappreciated clinical problem: Not only are a quarter of adults in the United States taking multiple (>3) medications [19], but >99% of physicians say they assess patients for DDIs, while often under-identifying whether an interaction has occurred [12]. Despite the ostensible availability and use of monitoring for DDIs through reconciliation, electronic health records, stewardships, etc., DECART2 shows again that the recognition of potentially severe/fatal DDIs is disturbingly low—especially amongst PCPs. Underscoring the severity of the problem is the fact that DDIs are iatrogenic, and high-quality care by definition means that we do not harm our patients. 

There are several limitations to this study: We used a subset of the original DECART1 cohort in the follow-up study; this may not be a concern, however, because we did not see any statistical difference between the physician characteristics among either the DECART1 or the DECART2 study participants. Similarly, although the intervention and control patients had statistically similar demographics and medical histories, we cannot account for any hidden heterogeneities in the two populations. The ubiquity of DDIs herein suggests that these results should possibly be translated into the routine use of DDI testing in patients taking more than four medications. When selecting patient eligibility, both intervention and control PCPs in this study chose from among three risk factors (i.e., non-compliance, unreported drug use, or other substance use) and two broad treatment areas (for behavioral health or chronic pain conditions), indicating that physicians were making educated guesses regarding patient risk for an interaction to occur. Further research might better demonstrate whether other conditions have as great and widespread non-compliance, substance use, and DDI-related issues as previously thought, requiring extra vigilance.

## 5. Conclusions

The results of DECART2 showed that the use of DDI testing in real-world patients significantly improved (1) the identification of potentially harmful drug interactions, (2) primary care patient management of drug interactions, and (3) patient outcomes. The study also shows that the use of CPV simulated patients to determine clinical utility is confirmed by real-world data.

## Figures and Tables

**Table 1:** Physician baseline characteristics.

	Control	Intervention	*p*-Value
*n*	21	21	
Male	76.1%	90.5%	0.214
*Age group*			
<40	9.5%	0.0%	0.294
40–55	42.9%	38.1%	
>55	47.6%	61.9%	
Internal medicine	28.6%	47.6%	0.204
Years in practice	21.0 + 8.7	24.6 + 4.8	0.103
Active patient panel	3328 + 1800	2893 + 1932	0.553
Receive quality bonus	57.1%	57.1%	1.000
*Region*			
Midwest	14.3%	28.6%	0.483
Northeast	23.8%	9.5%	
South	38.1%	42.9%	
West	23.8%	19.1%	
*Setting*			
Urban	28.6%	28.6%	0.911
Suburban	52.4%	57.1%	
Rural	19.1%	14.3%	
*Practice type*			
Private (solo)	19.1%	33.3%	0.546
Private (PCP only)	61.9%	47.6%	
Private (multispecialty)	19.1%	19.1%	
Other	0.0%	0.0%	
*CPV Scores*			
Diagnosis of DDI	21.6%	56.3%	<0.001
Discontinue substance causing DDI	26.6%	58.3%	<0.001
Necessary DDI-related treatment	15.9% + 17.4%	22.5% + 21.3%	0.001

**Table 2:** Patient characteristics.

	Control	Intervention	*p*-Value
Number of patients	169	162	n/a
Patient age	58.5	59.1	0.590
Male	52.7%	50.6%	0.710
Chronic conditions	64.5%	71.0%	0.207
Prescription medications	8.2	8.5	0.516
Physician-suspected risk factors			
Unclear compliance to prescription medications	72.2%	76.0%	0.699
Excessive alcohol/opioid consumption	50.3%	47.5%	0.615
Other interacting substances (e.g., supplements, OTC drugs, foods, etc.)	52.1%	54.9%	0.601

**Table 3:** Primary and secondary outcomes.

	Control	Intervention	*p*-Value
DDI-related recognition and treatment	7.1%	21.6%	<0.001
DDI noted on chart *	50.0%	45.7%	0.797
Counseling *	58.3%	48.6%	0.559
Stopping/adjusting interacting agent *	8.3%	62.9%	0.001
Resolution of symptoms *	20.1%	29.6%	0.045
Hospitalizations/ED visits *	11.2%	7.4%	0.231

* As a percentage of those who recognized a DDI.

## Data Availability

The data presented in this study are available on reasonable request from the corresponding author. The data are not publicly available due to privacy restrictions of research participants.

## Supplementary Materials

The following are available online at https://www.mdpi.com/article/10.3390/diagnostics11071266/s1: Table S1: List of substances detected by DDI testing.

## References

[1] Iyer S.V., Harpaz R., LePendu P., Bauer-Mehren A., Shah N.H. (2014). Mining clinical text for signals of adverse drug-drug interactions. J. Am. Med. Inform. Assoc..

[2] Magro L., Moretti U., Leone R. (2011). Epidemiology and characteristics of adverse drug reactions caused by drug-drug interactions. Expert Opin. Drug Saf..

[3] Bucşa C., Farcaş A., Cazacu I., Leucuta D., Achimas-Cadariu A., Mogosan C., Bojita M. (2013). How many potential drug-drug interactions cause adverse drug reaction in hospitalized patients?. Eur. J. Intern. Med..

[4] Arnold R.J., Tang J., Schrecker J., Hild C. (2018). Impact of Definitive Drug-Drug Interaction Testing on Medication Management and Patient Care. Drugs Real World Outcomes.

[5] Aspden P., Wolcott J.A., Bootman J.L., Cronenwett L.R. (2007). Preventing Medication Errors: Quality Chasm Series, The Institute of Medicine.

[6] Prevention of Adverse Drug Events in Hospitals. https://www.uptodate.com/contents/prevention-of-adverse-drug-events-in-hospitals.

[7] Sultana J., Cutroneo P., Trifiro G. (2013). Clinical and economic burden of adverse drug reactions. J. Pharmacol. Pharmacother..

[8] Prescription Drug Use in the Past 30 Days, by Sex, Race and Hispanic Origin, and Age: United States, Selected Years 1988–1994 through 2015–2018, Centers for Disease Control and Prevention. https://www.cdc.gov/nchs/hus/contents2019.htm#Table-039.

[9] Guthrie B., Makubate B., Hernandez-Santiago V., Dreischulte T. (2015). The rising tide of polypharmacy and drug-drug interactions: Population database analysis 1995–2010. BMC Med..

[10] Peabody J.W., Luck J., Glassman P., Jain S., Hansen J., Spell M., Lee M. (2004). Measuring the quality of physician practice by using clinical vignettes: A prospective validation study. Ann. Intern. Med..

[11] Peabody J.W., Luck J., Glassman P., Dresselhaus T.R., Lee M. (2000). Comparison of vignettes, standardized patients, and chart abstraction: A prospective validation study of 3 methods for measuring quality. JAMA.

[12] Peabody J., Acelajado M.C., Robert T., Hild C., Schrecker J., Paculdo D., Tran M., Jeter E. (2018). Drug-drug interaction assessment and identification in the primary care setting. J. Clin. Med. Res..

[13] Peabody J., Tran M., Paculdo D., Schrecker J., Valdenor C., Jeter E. (2018). Clinical utility of definitive drug-drug interaction testing in primary care. J. Clin. Med..

[14] Sertkaya A., Wong H.H., Jessup A., Beleche T. (2016). Key cost drivers of pharmaceutical clinical trials in the United States. Clin. Trials.

[15] Institute of Medicine (2014). Refining Processes for the Co-Development of Genome-Based Therapeutics and Companion Diagnostic Tests: Workshop Summary.

[16] Peabody J.W., Shimkhada R., Tong K.B., Zubiller M.B. (2014). New thinking on clinical utility: Hard lessons for molecular diagnostics. Am. J. Manag. Care.

[17] Peabody J., Rahim A., Wilcox B., McGehee C., Estigarribia E., Paculdo D., Arzadon A., Fugaro S., Tran M., Spitzer G. (2019). Clinical utility of a blood-based protein assay on diagnostic colonoscopy referrals for elevated-risk colorectal cancer patients in primary care. Am. J. Clin. Oncol..

[18] Peabody J., Tran M., Paculdo D., Valdenor C., Burgon T., Jeter E. (2019). Establishing clinical utility for diagnostic tests using a randomized controlled, virtual patient trial design. Diagnostics.

[19] Centers for Disease Control and Prevention. Fast Stats—Therapeutic Drug Use. https://www.cdc.gov/nchs/fastats/drug-use-therapeutic.htm.

