# Supplementary material for: Randomized Trial to Improve Primary Care Patient Management and Patient Outcomes Using a Drug–Drug Interaction Test: Confirmation of the DECART Simulated Patient Clinical Utility Trial Results
Source: Diagnostics (Basel). 2021 Jul 15;11(7):1266. doi: 10.3390/diagnostics11071266 (PMC8307579; doi:10.3390/diagnostics11071266)
Supplement: Supplementary file 1 [file diagnostics-11-01266-s001.zip › diagnostics-1288278-supplementary.pdf]

Supplementary Table S1. List of Substances Detected by DDI Testing*.

ANTIARRHYTHMICS
Amiodarone
Quinidine
Ranolazine
ANTIDEPRESSANTS AND ANTIPSYCHOTICS
Amitriptyline
Aripiprazole
Asenapine
Bupropion
Chlorpromazine
Citalopram/Escitalopram
Clomipramine
Desipramine
Desvenlafaxine
Doxepin
Duloxetine
Fluoxetine
Fluphenazine
Fluvoxamine
Haloperidol
Iloperidone
Nefazodone
Nortriptyline
Olanzapine
Paliperidone
Paroxetine
Perphenazine
Quetiapine
Risperidone
Sertraline
Thioridazine
Trazodone
Venlafaxine
ANTIEMETICS AND GASTRIC REFLUX
Cimetidine
Esomeprazole/Omeprazole
Famotidine
Lansoprazole
Metoclopramide
Ondansetron
Promethazine
Ranitidine
ANTIEPILEPTICS
Carbamazepine
Clobazam
Lamotrigine
Oxcarbazepine
Phenobarbital
Phenytoin
Primidone

ANTIHYPERTENSIVES
Amlodipine
Atenolol
Carvedilol
Diltiazem
Labetalol
Metoprolol
Nebivolol
Nifedipine
Propranolol
Timolol
Verapamil
ANTIMICROBIALS AND ANTIRETROVIRALS
Acyclovir/Valacyclovir
Atazanavir
Azithromycin
Chloroquine
Ciprofloxacin
Clarithromycin
Cobicistat
Darunavir
Delavirdine
Efavirenz
Erythromycin
Etravirine
Fluconazole
Fosamprenavir
Indinavir
Itraconazole
Ketoconazole
Levofloxacin/Ofloxacin
Metronidazole
Nelfinavir
Nevirapine
Posaconazole
Quinine
Rifabutin
Rifampin
Rifapentine
Ritonavir
Saquinavir
Terbinafine
Tipranavir
Voriconazole
ANTI-PARKINSON AGENTS
Carbidopa/Levodopa
Ropinirole
ANTITHROMBOTICS
Apixaban
Clopidogrel
Rivaroxaban
Warfarin
CHEMOTHERAPEUTIC AGENTS

Abiraterone
Anastrozole
Doxorubicin
Enzalutamide
Nilotinib
Pazopanib
COGNITIVE ENHANCEMENT
Atomoxetine
Donepezil
Guanfacine
Memantine
FOODS AND SUPPLEMENTS
Grapefruit Furanocoumarins
Kava
St. John's wort
INHALED CORTICOSTEROIDS AND BETA AGONISTS
Albuterol
Formoterol
Salmeterol
MISCELLANEOUS
Alcohol
Atorvastatin
Avanafil
Baclofen
Butalbital
Canagliflozin
Cyclobenzaprine
Hydroxyzine
Linagliptin
Lorcaserin
Methadone
Methocarbamol
Methotrexate
Mirabegron
Pioglitazone
Pseudoephedrine
Sumatriptan
Tizanidine
Zileuton
STEROIDS AND HORMONES
Dexamethasone
Methylprednisolone
Prednisone
*Complete list as provided to intervention physicians.
